# Reconstructive surgery for aortic valve stenosis

**DOI:** 10.1186/1749-8090-8-S1-O4

**Published:** 2013-09-11

**Authors:** Z Mitrev, T Anguseva, E Stoicovski, N Hristov, E Idoski

**Affiliations:** 1 Special Hospital for Surgery Fillip II, Skopje, Macedonia

## Background

The native aortic valve can be explained with rules of the equal side triangle, and as a part of the aortic root it is wedged between the heart and the ascending aorta. Beside different types of aortic valve replacements, reconstructive techniques are increasingly performed to restore normal aortic valve function. Reconstructive techniques themselves can be divided into isolated reconstruction of aortic valve/root structures and the isolated replacement of one or more structures. With this study we evaluated clinical results of reconstructive surgery of the aortic root with 3 leaflets pericardial patch.

## Methods

We created this reconstructive technique using bovine/equine pericardium, replacing valve cusps on aortic fibrous ring of patient. The leaflets are made from same pericardium from which other biologic valve prosthesis are done. The ring of patient’s aorta was used as guide for sizing this valve. Leaflets are implanted separately; using continuous sutures with 2 supported stitches at newly created commissure, without a stent or sowing ring. Main including criteria was stenosis of the aortic valve and patients with aortic annuli ring dilatation had been excluded. Intraoperative and postoperative TEE was performed for every created valve.

## Results

255 pts with aortic valvular disease had been included in study. 224 of them got bovine and 31 equine pericardium created leaflets. Middle aorta cross clamping time was 68min, and bypass time 105min. 35 patients got a aortocoronary bypass in combination (2.3 grafts per pts) 2 pts developed middle aortic regurgitation. Mortality rate was 4.4% (10 pts). Follow up period 1-132 months.

## Conclusions

Aortic root reconstructive surgery ensures hemodynamic improvement with a small transvalvular gradient in pts. It can be implanted even in patients with small root or with bicuspid valve, with good clinical outcome.

